# Supplementary material for: Ciclopirox Olamine Induces Proliferation Inhibition and Protective Autophagy in Hepatocellular Carcinoma
Source: Pharmaceuticals (Basel). 2023 Jan 12;16(1):113. doi: 10.3390/ph16010113 (PMC9863056; doi:10.3390/ph16010113)
Supplement: Supplementary file 1 [file pharmaceuticals-16-00113-s001.zip › pharmaceuticals-2118261-supplementary.pdf]

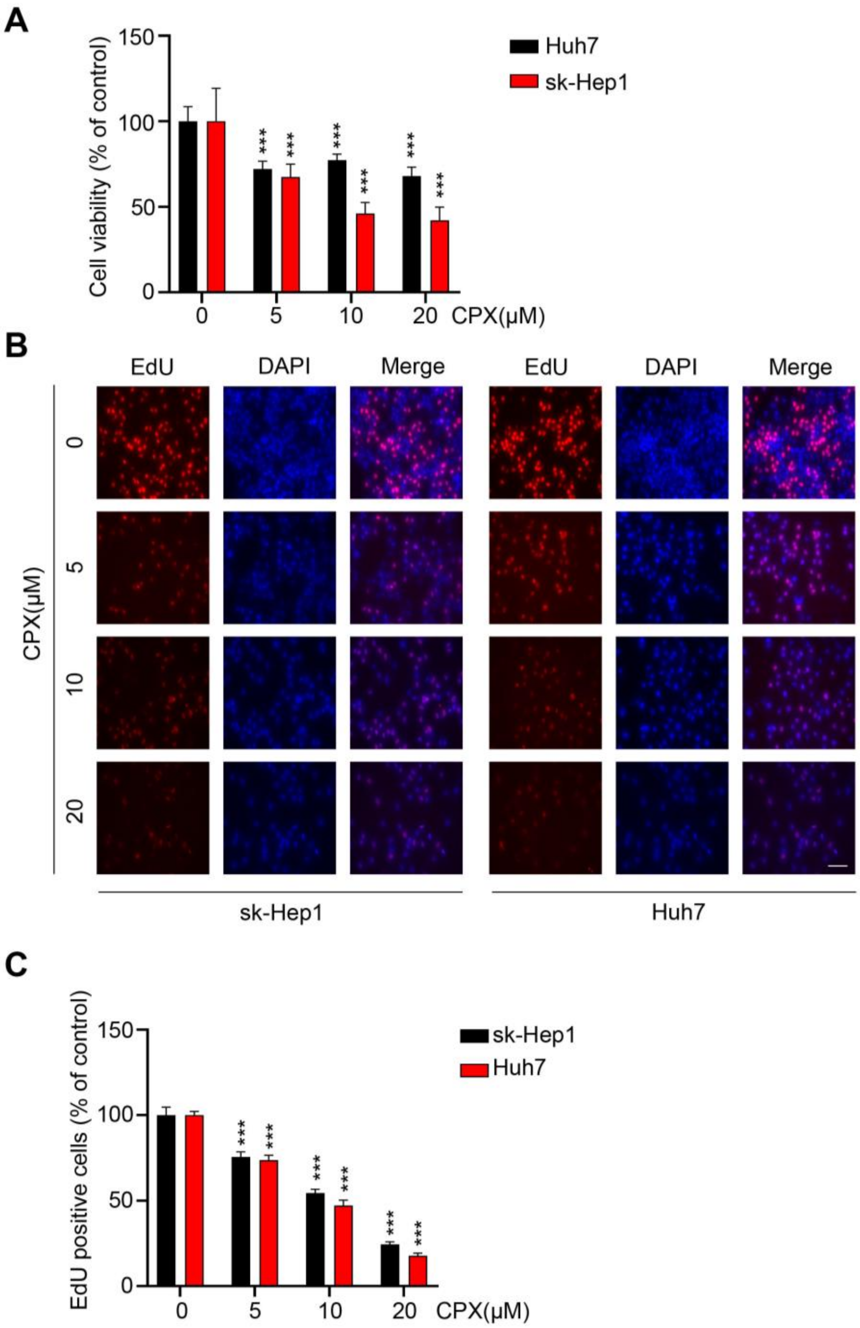

Figure S1. CPX inhibits proliferation in HCC cells. (**A**) The cell viability was detected by CCK8 kit. sk-Hep1 and Huh7 cells were treated with indicated concentrations of CPX for 48 h. (**B,C**) EdU assay of HCC cells treated with indicated concentrations of CPX for 48 h. The EdU positive cell rate was quantitated. Scale bar, 100 μm. Data are means ± s.d. and are representative of 3 independent experiments. ***, $p < 0.001$. Statistical significance compared with respective control groups.

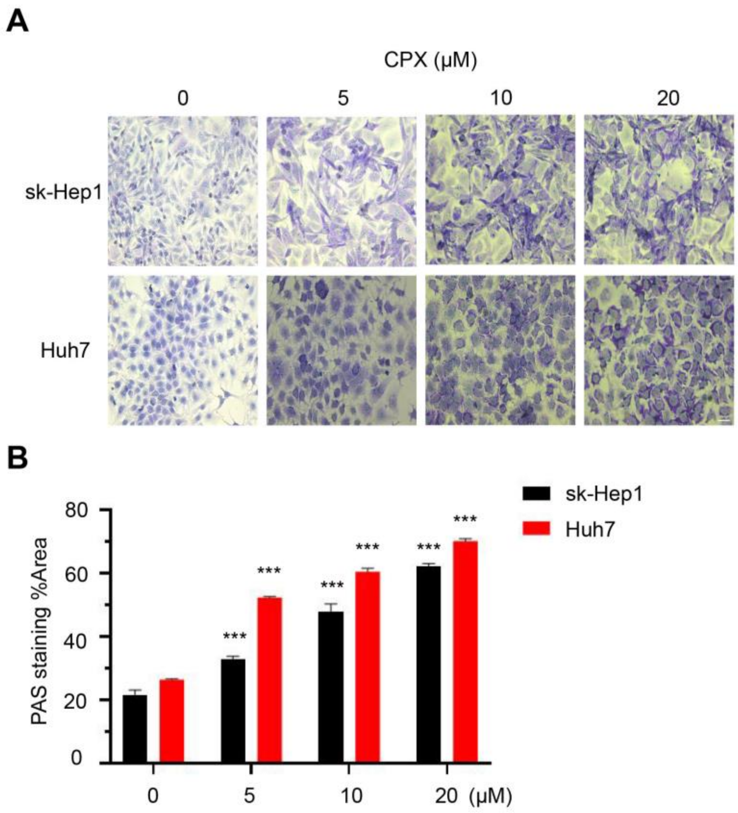

Figure S2. Glycogen clustering are induced by CPX in HCC cells. (**A**) PAS staining in sk-Hep1 and Huh7 cells treated with indicated concentrations of CPX for 24 h. (**B**) The PAS staining intensities of the images were quantified using Image J software. Scale bar, 50 μm. Data are means ± s.d. and are representative of 3 independent experiments. ***, $p < 0.001$. Statistical significance compared with respective control groups.
